# The influence of social network structures on leisure-time physical activity in hypertensive patients: a mixed-methods study in China

**DOI:** 10.1186/s12966-025-01845-1

**Published:** 2025-11-24

**Authors:** Bingjie Shen, Chenyang Pei, Tianjia Guan, Yuqing He, Ziqi He, Hui Li, Xiao Lu, Linghe Yang, Jinghong Zhao, Yuanli Liu

**Affiliations:** 1 School of Public Health, Bengbu Medical University, Bengbu, Anhui 233030 China; 2 School of Health Policy and Management, Chinese Academy of Medical Sciences & Peking Union Medical College, Beijing, 100005 China; 3 Faculty of Medicine and University Hospital, Heidelberg Institute of Global Health, Heidelberg University, Heidelberg, 69120 Germany; 4 School of Mental Health, Bengbu Medical University, Bengbu, Anhui 233030 China

**Keywords:** Leisure-time physical activity, Social network, Hypertension, Mixed-methods, Social context

## Abstract

**Background:**

Hypertension remains a major public health challenge in China. Leisure-time physical activity (LTPA) is critical for hypertension control. Yet its social network determinants, particularly key role models, are understudied in China’s familial-centric cultural context.

**Methods:**

This sequential mixed-methods study integrated quantitative surveys with qualitative interviews among hypertensive patients in Yichang, China. A total of 2639 patients were selected from 18 primary healthcare institutions using multi-stage random sampling method. Totally, 10,550 social relationships were nominated. Quantitative data on LTPA was measured by the International Physical Activity Questionnaire Long Form, and social network characteristics (size, density, LTPA aggregation, LTPA heterogeneity, and LTPA status of specific members) was gathered through the name generator method. Generalized additive mixed models assessed nonlinear associations between social network characteristics and LTPA levels (individual-level, *n = *2639); mixed-effects logistic regression analyzed member-patient LTPA linkages (relationship-level, *n = *10,550). Qualitative data (*n = *37) via interviews underwent grounded theory coding to contextualize mechanisms.

**Results:**

Among participants, 70.56% engaged in light intensity LTPA (L-LTPA), whereas 34.82% achieved ≥ 150 minutes/week moderate-to-vigorous intensity LTPA (MV-LTPA). Nonlinear thresholds were identified: smaller networks (≤ 4 members) with higher kinship density (≥ 65%) significantly increased MV-LTPA adherence (*P* < 0.001). Network-level LTPA aggregation demonstrated linearly positive with MV-LTPA (*P* < 0.001) but triphasic associations (i.e., increase then decrease to steady) with L-LTPA (*P* = 0.006). Critically, having a physically active spouse was the strongest predictor of MV-LTPA adherence (*OR* = 1.967, 95% *CI*: 1.571–2.463, *P* < 0.001). Qualitative themes revealed that kinship networks fostered LTPA through shared norms and social support.

**Conclusions:**

Social networks represent modifiable factors that influence LTPA behaviors. Integrating network-driven strategies into hypertension management and prioritizing spouses as “exercise advocates” could promote LTPA for middle-aged and elderly hypertensive patients in China. This study advances cross-cultural behavioral theory and offers actionable solutions for pragmatic solutions for global hypertension management in aging populations under similar social context.

## Introduction

### Challenges in hypertension management in China

Hypertension remains a critical public health crisis in China, characterized by two paradoxical trends: high prevalence (30.2% in men, 24.1% in women) yet alarmingly low control rates (13.9% in men, 17.8% in women) in 2019 [1]. This discrepancy persists despite the National Basic Public Health Service (BPHS) program since 2009, which mandates standardized hypertension management through primary care [2]. While BPHS has achieved some success in hypertension management, its impact on lifestyle modifications remains limited, which may explain suboptimal hypertension management outcomes [2].

### Dilemma of LTPA promotion in hypertension management

Physical activity (PA) is considered equally vital as pharmacological interventions in hypertensive management due to its benefits, cost-effectiveness, and minimal adverse effects [3–5]. The World Health Organization (WHO) explicitly recommends adherence to ≥ 150 min of moderate-to-vigorous intensity PA (MVPA) weekly as part of total PA for chronic disease populations [6, 7]. Concurrently, light intensity PA (LPA), though often less emphasized, holds significant value for this population. As a feasible entry point for sedentary individuals and a sustainable means to reduce prolonged sitting, LPA offers a complementary pathway to improve cardiovascular health, particularly for those who face barriers to engaging in MVPA [8–10]. Leisure-time physical activity (LTPA)—defined as voluntary exercise during non-working hours—is a critical pathway to achieve the WHO target, especially given declining occupational/household PA from urbanization [11, 12]. National surveys reveal progress yet persistent gaps: regular exercise (≥ 3 times/week and ≥ 30 min per time) rates has risen from 14.7% (2014) to 30.3% (2020) in adults [13, 14]; ≥ 150 min/week of moderate-to-vigorous intensity LTPA (MV-LTPA) remains below 25% [15, 16], with even lower rates among hypertensive patients due to obesity and safety concerns [16]. This also reflects a global challenge in translating clinical recommendations into sustained patient-driven behavioral change, underscoring the urgency of identifying novel and effective intervention strategies [17, 18].

### Evidence regarding social networks as a potential intervention lever for LTPA

Ecological models highlight multidimensional influences on LTPA [19], where social networks (the structural and functional architecture of interpersonal relationships [20]) represent an underexplored yet pivotal intervention lever, particularly within the Chinese context. In China, LTPA is often characterized by community-based activities (e.g., square dancing in public parks) and culturally embedded practices (e.g., tai chi), which are inherently social and deeply intertwined with the local relational fabric. This socio-cultural backdrop makes the influence of interpersonal networks on LTPA participation not only pronounced but also arguably inevitable, thereby necessitating a thorough investigation into its mechanisms.

Social networks shape health behaviors through structural (size, density, attributes of relationships, etc.) and functional (social support, resource access, social norms, behavioral contagion, etc.) dimensions according to Berkman’s theoretical framework [21] and growing evidence [22–26].

The *functions* provided by social networks play an proximal role in directly shaping behavioral outcomes [21, 22]. Among the existing functional mechanisms by which social networks influence LTPA, social contagion is an aspect that is often explored. Evidence demonstrats that network members' LTPA behaviors or exercise habits exert contagion effects [27]. Notably, studies suggest that individuals’ LTPA levels are positively associated not only with the actual behaviors of significant others but also with their perceptions of these individuals’ regular engagement in LTPA. Critically, such perceptions are found to exert a stronger influence on behaviors than objectively observed activity patterns [28].

However, these functions often depend on *structural* attributes and require precise structural metrics for evaluation. Crucially, social network research distinguishes itself by elucidating structural effects to guide interventions that modify network architecture [21, 22, 29]. Consequently, studies have explored the impact of objectively measurable structural indicators (e.g., network size, relational roles) on LTPA behaviors, grounded in functional mechanism theories. For instance, a Swiss study [30] demonstrated that individuals with regular exercise habits exhibited both larger proportions of active members and higher heterogeneity (balanced composition of active and inactive members) in their egocentric networks. Conversely, inactive individuals predominantly maintained networks with inactive members, often accompanied by discouraging interactions. Further analyses of relational attributes revealed familial clustering of PA levels in a Portuguese study [31], where kinship ties (spouses, parents, siblings) significantly shaped exercise habits. However, an adolescent study [32] highlighted peer networks (e.g., classmates or other friends of the same age) as dominant drivers of LTPA adoption.

### Research gaps and objectives: bridging cultural and methodological divides

Although evidence supports the potential intervention value of social networks in promoting LTPA, existing research exhibits critical gaps, resulting in the current BPHS protocol lacking culturally adapted strategies to leverage China’s kinship-driven social fabric for LTPA promotion.

#### First, contextual evidence

Limited evidence exists within the Chinese context. Current research remains Western-centric, emphasizing peer/community networks in interventions [30, 31]. In contrast, exercise behavior in China may be mediated through concentric kinship circles (spouses, siblings, multi-generational ties) under the "*differential mode of association* (*chaxugeju*)" social structure [33], rather than voluntary peer groups.

#### Second, study population

A critical gap persists in middle-aged and older adults with hypertension—a population epitomizing China’s kinship-centric social structure. Existing conclusions, predominantly derived from adolescent studies emphasizing peer/parental influences [32], lack generalizability to this demographic.

#### Third, predictor measurement

Prior studies disproportionately focus on individual-level metrics (e.g., network size) while neglecting relational roles (e.g., spouses, siblings) in shaping PA behaviors [29, 30]. Although evidence suggests differential behavioral impacts of relational roles [27, 31, 32], their specific pathways under China’s kinship hierarchy remain unclear. Identifying pivotal network influencers could optimize targeted behavioral interventions.

#### Fourth, outcome scope

Existing research on hypertensive patients has predominantly focused on MV-LTPA [16, 34], with scant attention paid to the patterns and correlates of light-intensity LTPA (L-LTPA). The influence of social networks on L-LTPA, in particular, remains virtually unexplored. This creates a significant knowledge gap regarding the social determinants of a more feasible and accessible form of PA, especially for patients who may be unable or unwilling to engage in MV-LTPA.

#### Additionally, analytical methods

Sociological variables demand refined analytical approaches. Current models often treat network features as continuous variables in linear regressions [29, 30], oversimplifying complex social mechanisms. Multidimensional statistical methods and advanced classification strategies are warranted to unravel nonlinear associations.

#### Moreover, research design

Underutilization of mixed-methods approaches limits holistic insights. Quantitative studies predominantly report statistical associations without contextualization, while qualitative works rely on subjective theorization lacking large-scale validation [35]. Integrating both methodologies could elucidate sociobehavioral mechanisms with enhanced rigor.

#### Finally, inconsistent findings

Inconsistent findings impede practical applications. For example, studies on social network size showed mixed results: positive [36], negative [37], or no association [30] with LTPA. Discrepancies may arise from cross-cultural variability, unidentified critical network factors, and methodological heterogeneity. Establishing mechanistic consensus requires transcultural studies employing diverse methodologies.

Thus, this study targets hypertensive patients in the Chinese social context and aims to identify network-driven mechanisms of both MV-LTPA and L-LTPA, informing culturally intelligent interventions for China’s BPHS system. We will address these gaps through: (1) Theoretical integration: Incorporating *differential mode of association theory* into ecological models to decode kinship network impacts on LTPA. (2) Mixed-methods design: Combining quantitative regression with qualitative interviews to quantify network-LTPA linkages and capture lived experiences of social network influence. (3) Threshold effect analysis: Identifying critical structural thresholds in social networks that influence LTPA outcomes. (4) Comparative intensity analysis: Examining L-LTPA alongside MV-LTPA to provide a more nuanced understanding of how social networks distinctly shape the entire spectrum of LTPA. (5) Intervention targeting: Pinpointing culturally salient network actors and effective support mechanisms to inform BPHS-aligned, network-enabled strategies.

## Methods

### Study design and setting

This sequential explanatory mixed-methods study combined cross-sectional surveys with semi-structured interviews from April 2021 to March 2023 in Yichang, China. Quantitative findings guided qualitative exploration, while interview narratives enriched statistical associations with real-life contexts and provided a mechanistic explanation for quantitative findings.

### Quantitative study

#### Participants and sampling

Inclusion criteria: (1) diagnosed with primary hypertension, without severe target organ damage; (2) hypertension management under the BPHS program for ≥ 1 year; (3) age 35–84 years; (4) permanent residency; and (5) able to complete the survey independently. Exclusion criteria: (1) severe complications (e.g., heart failure, stroke); (2) unable to complete the investigation due to cognitive/physical impairments; or (3) pregnancy.

**Fig. 1:**
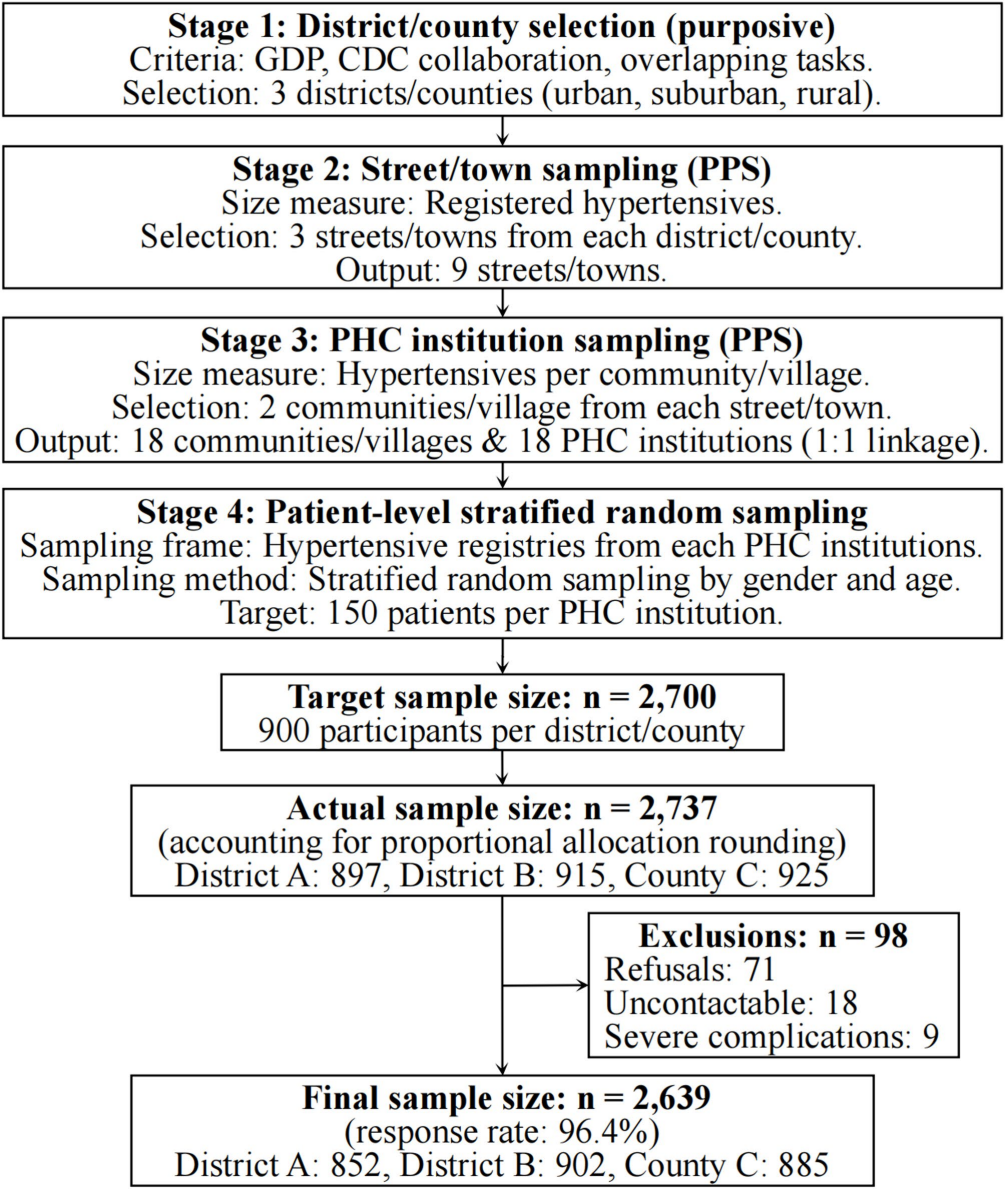
Four-stage stratified sampling framework. Abbreviations: GDP, Gross Domestic Product; CDC, Center for Disease Control; PPS, probability proportional to size; PHC, primary healthcare

Assuming a 50% prevalence of LTPA adherence (α = 0.05, margin of error = 5%, design effect = 2), the minimum sample size was calculated as 769 per district/county. We planned to recruit a total of 900 participants per district/county (accounting for 15% non-response), yielding a citywide target sample of 2,700. A four-stage stratified sampling design ensured geographic and demographic representativity (Fig. 1). After exclusions, a total of 2,639 participants remained.

#### Variables and measures

##### Outcomes: Leisure-Time Physical Activity (LTPA)

LTPA was assessed using the LTPA part of the International Physical Activity Questionnaire Long Form (IPAQ-LF). It has been used in Chinese population studies, and showed good validity and reliability [38]. We calculated the total duration of MV-LTPA as well as L-LTPA during the past week, equating one minute of vigorous intensity LTPA (V-LTPA) to two minutes of moderate intensity LTPA (M-LTPA). Referring to the WHO recommendation for total PA (150 min/week), we categorized MV-LTPA adherence into yes (≥ 150 min/week) and no (< 150 min/week) groups. We categorized L-LTPA engagement into yes (> 0 min/week) and no (0 min/week) groups to capture the critical threshold of any L-LTPA engagement that disrupts sedentarism. Since WHO recommended PA for any length of time was encouraged, we removed the requirement that the traditional IPAQ only measure PA exceeding 10 min in a single session. We also found the results were similar to those when less than 10 min PA were excluded.

##### Explanatory variables: egocentric social network structural characteristics

Egocentric social network data were collected via a questionnaire employing the name generator method, a widely used tool for egocentric network analysis [39, 40]. Participants were asked to list contacts who frequently interacted with them or provided support relevant to hypertension self-care over the previous six months. Support types included informational (health advice), emotional (comfort during discomfort), instrumental (financial assistance for healthcare), and caregiving support. Participants could name up to seven members/alters per category across five questions, yielding a maximum of 35 network members. For each listed member, participants reported their relationship (e.g., spouse/partner, children, parents, siblings, neighbor, colleague, friend) and whether the member was physically active based on participants’ perception of member’s LTPA levels. Participants were provided with the 150 min/week MV-LTPA benchmark as a reference for their judgments. Members with ≥ 150 min/week MV-LTPA were classified as physically active; others (including cases with unknown LTPA levels) were deemed physically inactive. The structural network characteristics were computed from the name generator data at two levels: individual and relational. Detailed information of explanatory variables were provided in Table 1.

**Table 1:** Explanatory variables

Characteristics	Description or calculation formula	Range and interpretation
Individual-level		
Basic structure		
Network size	Number of social relationships or social network members that the patient mentioned in the questionnaire	Integer within [0, 35]: Larger values indicate a larger network
Network density	Proportion of strong ties (close relatives) in the network. Close relatives are defined according to China’s social structure and the Civil Code, including spouses, parents, children, siblings, grandparents, and grandchildren	[0, 100%]: Larger values indicate a denser network
LTPA-related structure		
LTPA aggregation	Proportion of physically active network members relative to the total number of network members	[0, 100%]: Larger values indicate higher aggregation of physically active members
LTPA heterogeneity	IQV = $\frac{\text{1} - \sum {\text{Pi}}^{2}}{\text{1} - \frac{1}{{\text{r}}}}$, where *Pi* is the proportion of physically active members or inactive members, and *r* is the number of variable categories	[0, 1]: Larger values indicate greater heterogeneity (more balanced proportions of active/inactive members)
Relationship-level		
LTPA of network members of specific relationship	Whether a network member was physically active, which was identified based on the participant’s subjective perception	0 = Inactive; 1 = Active

*Abbreviations*: *LTPA* Leisure-time physical activity, *IQV* Index of Qualitative Variation

##### Covariates

Covariates were selected a priori based on previous literature and biological plausibility, including sociodemographics (gender, age, residence, education, occupation, income and marital status) and health status (body mass index [BMI] and self-rated health). These were collected using structured questionnaires during face-to-face interviews.

#### Data collection and quality control

Trained health workers conducted face-to-face interviews using structured questionnaires. Rigorous quality checks were implemented pre-, during, and post-data collection.

#### Statistical methods

The analytical strategy comprised descriptive statistics, followed by a two-level regression framework. All statistical analyses were performed using R 4.5.1. The Bonferroni correction was used to account for the multiple comparisons.

##### Individual-level analysis: nonlinear associations (*n = *2639 patients)

Generalized additive mixed models (GAMMs) were implemented to jointly address nonlinear relationships between individual-level network metrics and LTPA outcomes while accounting for the hierarchical data structure (patients nested within 18 healthcare institutions). A Gaussian random intercept for the healthcare institution (k = 18) was incorporated to capture clustering effects, justified by significant intraclass correlation coefficient (*ICC*) = 0.305 for the MV-LTPA. Random effects at the district/county level were excluded due to limited group counts (k = 3), which could lead to biased variance estimates. Instead, fixed effects included residence (urban/suburban/rural, categorical covariate to adjust for regional disparities) as well as standard confounders (gender, age, education, etc.). Penalized B-splines (P-splines) were chosen for their robustness to sparse and skewed data, utilizing second-order difference penalties to control smoothness, which is not as sensitive to knot numbers and placements as restricted cubic splines [41]. Smooth terms were estimated via fast restricted maximum likelihood (fREML) optimization, which accelerates computation for large datasets while maintaining estimation accuracy comparable to conventional REML. The maximum basis dimension for each spline was set to k = 10 to accommodate potential complexity, with optimal smoothing parameters determined data-adaptively through fREML score minimization. Model validity was assessed through: (1) quantile–quantile plots of residuals to verify approximate normality; and (2) sensitivity analyses comparing P-splines to thin-plate splines. Models were fitted using the bam() function (Bayesian generalized additive models for large datasets) in the R package mgcv. For metrics exhibiting significant nonlinearity, inflection points were identified via first-derivative analysis of GAMM smoothing curves, and segments were defined ensuring adequate sample size in each to guarantee stable effect estimation with multiple covariates. Furthermore, segment-specific associations were quantified using piecewise mixed-effects logistic regression.

##### Relationship-level analysis: influential ties (*n = *10,550 ties)

To identify which specific types of network members (e.g., spouses, friends) were associated with patients' LTPA, we conducted an analysis at the relationship level. Given the multilevel structure (ties nested within participants, participants nested within institutions), we fitted stratified mixed-effects logistic regression models for each of the 13 relationship subgroups. Each model included a binary outcome (participant’s LTPA outcomes: yes/no) and adjusted for three key dimensions: (1) core predictor: tie member’s LTPA status (active/inactive), (2) participant-level covariates: gender, age, residence, occupation, income, marital status and self-rated health, and (3) institution-level random intercept: to account for clustering of participants within the 18 recruitment institutions. To ensure model stability in smaller subgroups, covariates were refined by consolidating age and self-rated health into ordinal variables (≤ 3 levels) and excluding non-significant variables (education, BMI) from the participant-level analysis

### Qualitative study

#### Participant selection

A purposive sampling strategy (maximum variation) was employed to select 37 participants from the quantitative study population to ensure diversity in age, gender, residence, and LTPA levels. Sampling continued until thematic saturation was achieved.

#### Data collection

Semi-structured interviews were conducted to explore social network characteristics and facilitators/barriers to exercise (using the term "exercise" instead of "LTPA" for clarity). All interviews were audio-recorded, transcribed verbatim, and cross-verified for accuracy.

#### Thematic analysis

Thematic analysis was guided by grounded theory principles. This involved an iterative process of: (1) open coding: iterative categorization of raw data into descriptive codes; (2) axial coding: grouping codes into thematic categories; and (3) selective coding: synthesizing categories into overarching themes. Analytical rigor was ensured through analyst triangulation and member checking.

## Results

### Quantitative results

#### Sociodemographic, physical-health, social network structural characteristics and LTPA levels of participants

A total of 2,639 hypertensive patients were included, with a median age of 65 years (interquartile range [*IQR*]: 58–70). The detailed sociodemographic and physical-health characteristics of the participants are presented in Table 2.

**Table 2:** Baseline characteristics of the study participants (*n = *2639)

Characteristics	Overall (proportion, %)	MV-LTPA adherence (rate, %)	*P* value
Total	2639 (100.00)	919 (34.82)	
**Sociodemographic characteristics**			
Gender			0.009
Male	1217 (46.12)	392 (32.21)	
Female	1422 (53.88)	527 (37.06)	
Age (Years)			0.019
35–	88 (3.33)	23 (26.14)	
45–	343 (13.00)	100 (29.15)	
55–	800 (30.31)	273 (34.13)	
65–	1313 (49.75)	491 (37.40)	
75–84	95 (3.61)	32 (33.68)	
Residence			< 0.001
Urban	852 (32.28)	434 (50.94)	
Suburban	902 (34.18)	332 (36.81)	
Rural	885 (33.54)	153 (17.29)	0.009
Education level			
Less than primary school graduation	518 (19.63)	156 (30.12)	
Primary school graduation	805 (30.50)	265 (32.92)	
Junior high school graduation	868 (32.89)	330 (38.02)	
High school graduation or above	448 (16.98)	168 (37.50)	< 0.001
Occupation			
Salaried employment	365 (13.83)	124 (33.97)	
Farmer	1158 (43.88)	257 (22.19)	
Unemployed	1116 (42.29)	538 (48.21)	< 0.001
Personal monthly income (yuan)			
0–	917 (34.75)	255 (27.81)	
1000–	766 (29.03)	267 (34.86)	
2000–	434 (16.45)	185 (42.63)	
3000–	294 (11.14)	134 (45.58)	
4000–	228 (8.63)	78 (34.21)	0.007
Marital status			
Non-married	38 (1.44)	8 (21.05)	
Married	2240 (84.88)	760 (33.93)	
Divorce/separation	52 (1.97)	21 (40.38)	
Widowed	309 (11.71)	130 (42.07)	
**Physical-health characteristics**			< 0.001
Self-rated health			
Very poor	74 (2.80)	15 (20.27)	
Poor	352 (13.34)	77 (21.88)	
Fair	1113 (42.18)	436 (39.17)	
Good	910 (34.48)	349 (38.35)	
Excellent	190 (7.20)	42 (22.11)	0.411
BMI			
Underweight	47 (1.78)	12 (25.53)	
Normal weight	1104 (41.83)	380 (34.42)	
Overweight	1075 (40.74)	388 (36.09)	
Obesity	413 (15.65)	139 (33.66)	
Social network structural characteristics^a^			
Network size (person)	4 (2–5)	3 (2–5)	< 0.001
Network density (%)	77.78(66.67–100.00)	100.00(66.67–100.00)	< 0.001
LTPA aggregation (%)	50.00(25.00–71.43)	50.00(33.33–100.00)	< 0.001
LTPA heterogeneity	0.75(0.00–0.94)	0.75(0.00–0.89)	< 0.001

*Abbreviations*: *LTPA* Leisure-time physical activity, *MV-LTPA* Moderate-to-vigorous intensity leisure-time physical activity, *BMI* Body mass index

^a^The continuous variables with non-normal distribution were described by the median (lower quartile–upper quartile)

During the past week, 70.56% (*n = *1862) of hypertensive patients engaged in L-LTPA for a median of 4 (*IQR*: 3–6) days/week, totaling 90 (*IQR*: 45–180) minutes/week. Moreover, 52.71% (1391) participated in M-LTPA for 3 (*IQR*: 2–5) days/week, totaling 180 (*IQR*: 60–360) minutes/week. Additionally, 32.21% (850) engaged in V-LTPA for 1 (*IQR*: 1–2) days/week, totaling 60 (*IQR*: 30–90) minutes/week. Overall, 63.77% (1683) engaged in MV-LTPA and 34.82% (919) met ≥ 150 min/week of MV-LTPA.

A total of 10,550 social network members were nominated by the 2639 hypertensive patients. Among these ties, 69.06% involved close relatives, with distant relatives (16.86%) and friends (14.08%) comprising the remainder. At the individual level (*n = *2639), the median network size was 4 persons, ranging from 1 to 11. Notably, patients with ≥ 150 min/week of MV-LTPA had smaller networks but higher density and greater LTPA aggregation (Table 2).

#### Associations of sociodemographic and physical-health characteristics with LTPA

Table 3 identified significant sociodemographic and physical-health characteristics for LTPA outcomes. For MV-LTPA, lower odds were observed in males (vs. females), adults aged 75–84 years (vs. 65–), rural residents (vs. urban), salaried employment or farmers (vs. unemployed), and those reporting very poor, poor or excellent self-rated health (vs. moderate). Higher odds of MV-LTPA occurred in participants with monthly income of 2000– yuan (vs. 0–) and widowed individuals (vs. married). For L-LTPA, only farmers (higher odds) and very poor self-rated health (lower odds) showed significant associations. All *P* < 0.05.

**Table 3:** Associations of sociodemographic and physical-health characteristics with LTPA (*n = *2639)

Characteristics	*OR* [95% *CI*] for MV-LTPA adherence	*P* value	*OR* [95% *CI*] for L-LTPA engagement	*P* value
**Sociodemographic characteristics**				
Gender				
Male	0.735 [0.600–0.900]	0.003	0.851 [0.699–1.035]	0.106
Female	Ref		Ref	
Age (Years)				
35–	0.745 [0.413–1.343]	0.328	1.695 [0.952–3.019]	0.073
45–	0.938 [0.658–1.336]	0.721	1.362 [0.958–1.937]	0.085
55–	1.162 [0.920–1.467]	0.207	0.968 [0.771–1.214]	0.778
65–	Ref		Ref	
75–84	0.423 [0.256–0.698]	0.001	1.335 [0.810–2.200]	0.256
Residence				
Urban	Ref		Ref	
Suburban	0.674 [0.239–1.898]	0.455	0.844 [0.426–1.674]	0.628
Rural	0.202 [0.070–0.584]	0.003	1.892 [0.933–3.836]	0.077
Education level				
Less than primary school graduation	Ref		Ref	
Primary school graduation	0.930 [0.702–1.231]	0.611	1.006 [0.764–1.323]	0.969
Junior high school graduation	1.023 [0.755–1.386]	0.885	0.906 [0.675–1.216]	0.510
High school graduation or above	0.880 [0.606–1.279]	0.503	1.074 [0.747–1.545]	0.700
Occupation				
Salaried employment	0.697 [0.491–0.989]	0.043	1.296 [0.919–1.828]	0.140
Farmer	0.681 [0.508–0.912]	0.010	1.535 [1.165–2.023]	0.002
Unemployed	Ref		Ref	
Personal monthly income (yuan)				
0–	Ref		Ref	
1000–	1.222 [0.938–1.592]	0.138	0.861 [0.668–1.111]	0.249
2000–	1.432 [1.026–1.999]	0.035	0.870 [0.627–1.206]	0.403
3000–	1.229 [0.825–1.830]	0.311	0.801 [0.548–1.170]	0.252
4000–	1.276 [0.814–2.000]	0.288	0.721 [0.472–1.102]	0.130
Marital status				
Non-married	0.932 [0.392–2.219]	0.874	0.691 [0.329–1.451]	0.329
Married	Ref		Ref	
Divorce/separation	1.555 [0.824–2.934]	0.173	1.476 [0.759–2.871]	0.252
Widowed	1.439 [1.072–1.932]	0.015	1.071 [0.799–1.435]	0.648
**Physical-health characteristics**				
Self-rated health				
Very poor	0.507 [0.269–0.954]	0.035	0.320 [0.191–0.538]	< 0.001
Poor	0.628 [0.458–0.862]	0.004	0.793 [0.594–1.059]	0.116
Fair	Ref		Ref	
Good	0.870 [0.700–1.082]	0.211	1.230 [0.991–1.528]	0.061
Excellent	0.524 [0.340–0.809]	0.004	1.343 [0.867–2.080]	0.187
BMI				
Underweight	0.885 [0.410–1.909]	0.755	0.712 [0.356–1.424]	0.337
Normal weight	Ref		Ref	
Overweight	1.007 [0.822–1.234]	0.945	1.056 [0.865–1.288]	0.592
Obesity	1.108 [0.841–1.459]	0.467	0.887 [0.681–1.155]	0.373

*Abbreviations*: *MV-LTPA* Moderate-to-vigorous intensity leisure-time physical activity, *L-LTPA* Light intensity leisure-time physical activity, *OR* Odds Ratio, 95% *CI* 95% Confidence Interval, *BMI* Body mass index

#### Dose–response relationships between social network metrics and LTPA outcomes

**Fig. 2:**
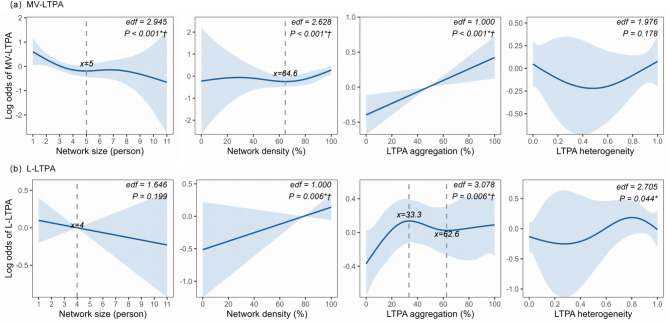
Nonlinear associations between social network metrics and LTPA outcomes (individual-level, *n = *2639). The figure depicts the relationships of network size, density, LTPA aggregation, and LTPA heterogeneity with the log odds of (**a**) achieving ≥ 150 minutes/week of MV-LTPA and (**b**) engaging in L-LTPA. Solid lines: Penalized spline fits from GAMMs; Shaded areas: 95% *CI*; Dashed vertical lines: Inflection thresholds identified where first derivative = 0 and segment sample size ≥ 10 times the number of predictors. All models adjusted for gender, age, residence, education, occupation, income, marital status, BMI, self-rated health and institution-level random effects. Statistical annotations: edf, effective degrees of freedom; * *P* < 0.05; † *P* < 0.00625 (Bonferroni-corrected significance). Abbreviations: LTPA, leisure-time physical activity; MV-LTPA, moderate-to-vigorous intensity leisure-time physical activity; L-LTPA, light intensity leisure-time physical activity; IQV: Index of Qualitative Variation; GAMMs, Generalized additive mixed models; 95% *CI*, 95% Confidence Interval; BMI, Body mass index

Figure 2 and Table 4 revealed distinct nonlinear patterns between social network metrics and different intensities of LTPA.

**Table 4:** Linear and segment-specific associations between social network metrics and LTPA outcomes (*n = *2639)

Variables	Linear model *OR* [95% *CI*]	*P* value	Segmented model *OR* [95% *CI*]	*P* value	Δ AIC
Outcome: MV-LTPA adherence					
Network size (per 1 increase)	0.905 [0.858–0.954]	< 0.001	1–: 0.826 [0.756–0.903]	< 0.001	−4.4 *P* = 0.012
			5–11: 1.035 [0.923–1.161]	0.558	
Network density (per 10% increase)	1.103 [1.050–1.159]	< 0.001	0–: 0.906 [0.788–1.041]	0.165	
			64.6–100: 1.191 [1.108–1.279]	< 0.001	−6.2 *P* = 0.004
LTPA aggregation (per 10% increase)	1.086 [1.055–1.118]	< 0.001			
LTPA heterogeneity (per 0.1 increase)	0.997 [0.976–1.019]	0.808			
Outcome: L-LTPA engagement					
Network size (per 1 increase)	0.968 [0.921–1.017]	0.200			
Network density (per 10% increase)	1.068 [1.019–1.119]	0.006			
LTPA aggregation (per 10% increase)	1.031 [1.002–1.061]	0.033	0–: 1.233 [1.117–1.361]	< 0.001	−9.9 *P* < 0.001
			33.3–: 0.878 [0.780–0.988]	0.030	
			62.6–100:1.058 [0.970–1.153]	0.201	
LTPA heterogeneity (per 0.1 increase)	1.022 [1.001–1.044]	0.044			

All models adjusted for gender, age, residence, education, occupation, income, marital status, BMI, self-rated health and institution-level random effects. Δ AIC = difference vs. linear model

*Abbreviations*: *MV-LTPA* Moderate-to-vigorous intensity leisure-time physical activity, *L-LTPA* Light intensity leisure-time physical activity, 95% *CI* 95% Confidence Interval, *OR* Odds Ratio, *BMI* Body mass index, *AIC* Akaike Information Criterion

Network size exhibited a threshold effect for MV-LTPA adherence (effective degrees of freedom [edf] = 2.945, *P* < 0.001), characterized by a sharp decline in odds as size increased to approximately 5 ties (Odds Ratio [*OR*] = 0.826, 95% Confidence Interval [*CI*]: 0.756–0.903), beyond which the effect stabilized. In contrast, its association with L-LTPA engagement was a non-significant linear decrease (edf = 1.646, *P* = 0.199).

Network density displayed a delayed-onset relationship with MV-LTPA (edf = 2.628, *P* < 0.001), where the positive effect remained weak until density exceeded about 64.6%, after which it increased sharply (*OR* = 1.191 per 10% density increase, 95% *CI*: 1.108–1.279). Whereas for L-LTPA it demonstrated a steady linear increase (edf = 1.000, *P* = 0.006; *OR* = 1.068 per 10% density increase, 95% *CI*: 1.019–1.119).

LTPA aggregation (the proportion of physically active members in one's network) showed a straightforward, linear increase in the odds of meeting ≥ 150 min/week of MV-LTPA (edf = 1.000, *P* < 0.001; *OR* = 1.086 per 10% aggregation increase, 95% *CI*: 1.055–1.118). However, its relationship with L-LTPA was more complex and triphasic (i.e., having three distinct phases, edf = 3.078, *P* = 0.006). The odds of L-LTPA initially increased as the proportion of active members rose, reaching a peak benefit at around one-third (33.3%) of members being active (*OR* = 1.233 per 10% aggregation increase, 95% *CI*: 1.117–1.361). Beyond this peak, the benefit began to diminish, eventually declining to a lower level by the time about 62.6% of members were active (*OR* = 0.878 per 10% aggregation increase, 95% *CI*: 0.780–0.988). After that, the effects stabilized.

After adjusting for multiple comparisons, LTPA heterogeneity (the diversity of LTPA levels of members) within a network did not show statistically significant associations with either MV-LTPA or L-LTPA (MV-LTPA: edf = 1.976, *P* = 0.178; L-LTPA: edf = 2.705, *P* = 0.044).

#### Associations between tie members’ LTPA and participants’ LTPA outcomes across different relationships

**Fig. 3:**
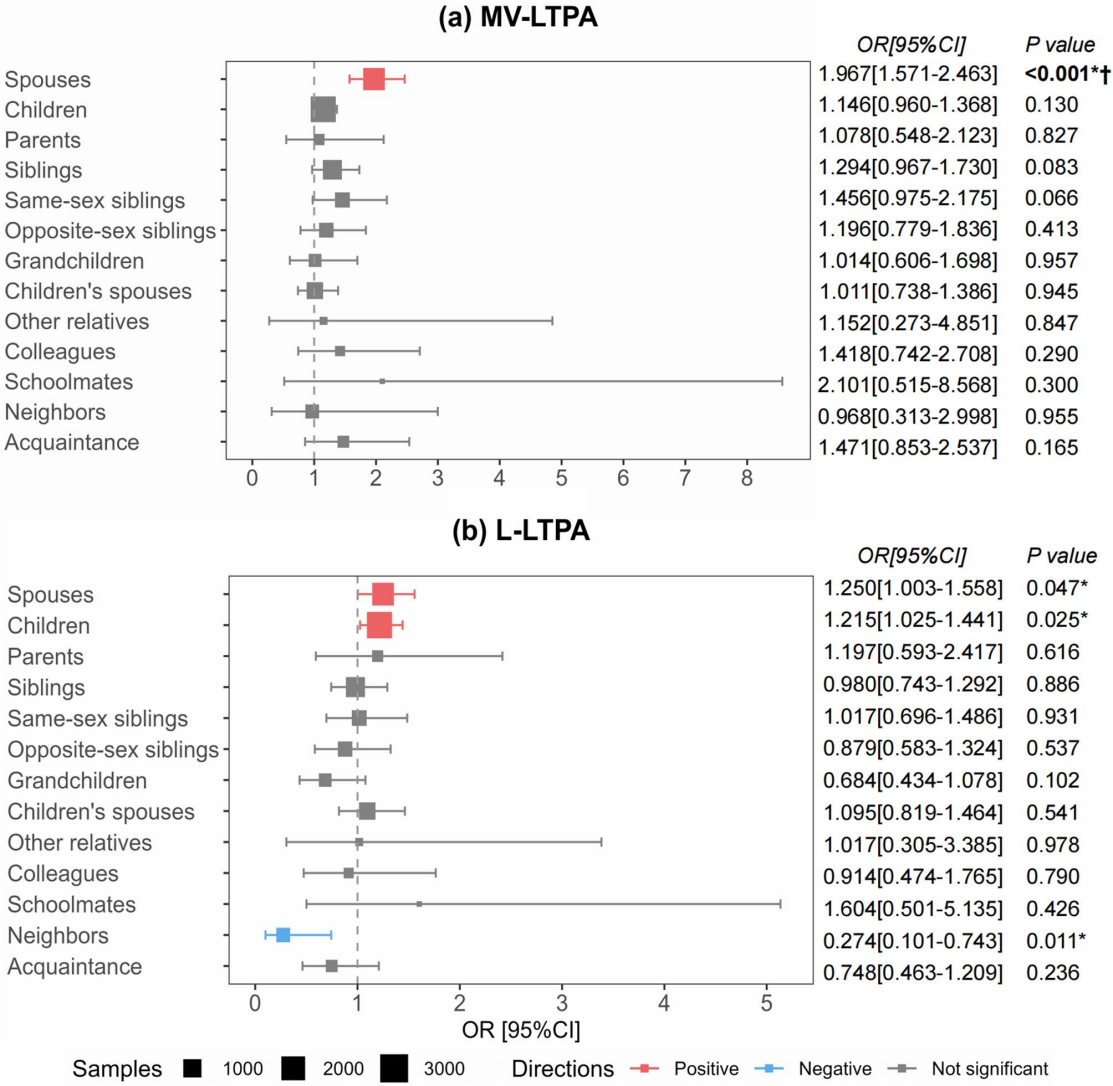
Forest plots of relationship-specific associations between tie members' LTPA and participant’ LTPA outcomes (relationship-level, *n* = 10,550). **a** Odds ratios for achieving ≥ 150 minutes/week of MV-LTPA. **b** Odds ratios for engaging in L-LTPA. Error bars represent 95% CI. Estimates derived from stratified mixed-effects logistic regression models with covariates (gender, age, residence, occupation, income, marital status and self-rated health) and institution-level random intercepts. Statistical annotations: * Uncorrected *P* < 0.05; † Significant after Bonferroni correction for 13 subgroups. Abbreviations: LTPA, leisure-time physical activity; MV-LTPA, moderate-to-vigorous intensity leisure-time physical activity; L-LTPA, light intensity leisure-time physical activity; OR, Odds Ratio; 95% CI, 95% Confidence Interval

Stratified mixed-effects logistic regression analyses revealed heterogeneous effects across relationship types (Fig. 3). For participants’ MV-LTPA adherence, spouses' LTPA showed the strongest positive association (*OR* = 1.967, 95% *CI*: 1.571–2.463, *P* < 0.001), remaining statistically significant after Bonferroni correction for 13 subgroups. In contrast, no other relationship type significantly predicted MV-LTPA adherence post-correction. For participants' L-LTPA engagement, spouses' LTPA again demonstrated a positive association (*OR* = 1.250, 95% *CI:* 1.003–1.558, *P* = 0.047). Similarly, children's LTPA was positively associated with L-LTPA engagement (*OR* = 1.215, 95% *CI*: 1.025–1.441, *P* = 0.025). Notably, neighbors' LTPA exhibited an inverse relationship with L-LTPA engagement (*OR* = 0.274, 95% *CI*: 0.101–0.743, *P* = 0.011). However, these three associations with L-LTPA did not survive multiple testing correction. All models accounted for participant covariates and institutional clustering effects.

### Qualitative results: social network facilitators and barriers to exercise

The participants in this study were predominantly elderly patients. Their social networks were generally small in scale and structurally homogeneous, with spouses as the core members and a minority involving close family members such as children, children-in-law, and grandchildren. Their daily social engagement was notably limited. This phenomenon can be attributed to multidimensional factors, including: intergenerational separation (e.g., long-term migration of children for employment), time constraints due to household or labor burdens, caregiving responsibilities (e.g., grandchild care), health-related limitations, and a low willingness to socialize (e.g., introversion or deliberate avoidance of interactions).

Grounded theory analysis revealed two themes for social network facilitators: (1) Positive social norms and interactions from network members supporting exercise (e.g., joint workouts, exercise invitations). (2) Social support provided by network members, including informational (sharing exercise benefits), emotional (encouragement), and instrumental support (equipment provision). One theme for social network barriers was identified: Negative social norms and interactions among network members that discourage exercise (e.g., perceptions of exercise as exhausting, or invitations for sedentary activities). Coding details are presented in Table 5.

**Table 5:** Social network facilitators and barriers to exercise

Direction	Theme	Category	Coding
1.Facilitators	1.1. Positive social norms and interactions among network members supporting exercise	1.1.1. Attitudes of network members supporting exercise	1.1.1.1. Network members perceive not exercising as an unhealthy lifestyle
		1.1.2. Behaviors of network members supporting exercise	1.1.2.1. Other members in the network exercise regularly
			1.1.2.2. Network members exercise around the patient
		1.1.3. Positive interactions between exercising network members and the patient	1.1.3.1. The patient lives with exercising network members
			1.1.3.2. The patient exercises with network members
			1.1.3.3. Network members invite the patient to exercise together
	1.2. Social support provided by network members	1.2.1. Informational support	1.2.1.1. Network members share personal health benefits gained from exercise
			1.2.1.2. Network members share that exercise helps control blood pressure
			1.2.1.3. Network members offer suggestions on how to exercise
		1.2.2. Emotional support	1.2.2.1. Network members show concern for health issues related to exercise
			1.2.2.2. Network members encourage the patient to exercise actively
			1.2.2.3. Network members provide care when the patient feels sad and does not want to exercise
		1.2.3. Instrumental support	1.2.3.1. Network members buy exercise equipment for the patient
			1.2.3.2. Network members remind the patient to participate in exercise
2. Barriers	2.1. Negative social norms and interactions among network members that discourage exercise	2.1.1. Negative attitudes of network members towards exercise	2.1.1.1. Network members express dissatisfaction with exercise, believing it to be too tiring
		2.1.2. Behaviors of network members not supporting exercise	2.1.2.1. Other members in the network do not exercises
			2.1.2.2. Network members do not exercise around the patient
		2.1.3. Negative interactions between non-exercising network members and the patient	2.1.3.1. The patient lives with non-exercising network members
			2.1.3.2. The patient spends leisure time with sedentary network members
			2.1.3.3. The patient engages in sedentary recreational activities with network members
			2.1.3.4. Network members invite the patient to engage in sedentary recreational activities

## Discussion

### MV-LTPA levels and associated determinants

This multi-stage random sampling study of middle-aged and elderly hypertensive patients in Yichang City revealed that 70.56% engaged in L-LTPA, with 34.82% meeting ≥ 150 min/week of MV-LTPA. While this exceeds rates reported in general populations by prior studies [15, 16], it remains substantially lower than figures from developed nations (e.g., 38.2%–50.4% in the US [42]). This disparity highlights contextual differences, in which occupational and domestic activities dominate physical exertion in China, whereas leisure-based activities prevail in Western settings.

Critically, approximately two-thirds of hypertensive patients still fell short of LTPA levels, underscoring an urgent need for targeted interventions. MV-LTPA adherence varied across subgroups. Males exhibited lower MV-LTPA than females, likely due to gendered activity patterns (e.g., work-related exertion vs. household or leisure activities) [15, 43]. Rural residents, agricultural workers and employed individuals also demonstrated poorer MV-LTPA outcomes, aligning with previous studies [15, 42, 44]. These findings emphasize socioeconomic and cultural barriers to LTPA, particularly among vulnerable populations. This study found that married patients exhibited significantly lower MV-LTPA compared to widowed individuals. Evidence indicated that both supportive and stressful marital dynamics could enhance LTPA, suggesting that marital quality may be a more critical determinant rather than mere marital status [45]. Additionally, the observed lower MV-LTPA levels among individuals with poorer self-rated health aligns with the findings of Han [46], though reverse causality warrants caution (e.g., higher LTPA may improve self-rated health through its benefits). Notably, even individuals with excellent self-rated health demonstrated relatively low MV-LTPA levels—a previously unreported phenomenon potentially attributable to exercise neglect driven by overconfidence in health status.

### Social network influence on MV-LTPA

#### Basic structural characteristics

Hypertensive patients predominantly maintained small, kinship-centered networks, consistent with Fei Xiaotong’s *differential mode of association* theory [33]. A nonlinear association emerged for MV-LTPA, whereby patients with extremely small and tightly-knit core networks were more likely to achieve higher MV-LTPA. This finding expands the theoretical boundaries of network-health behavior relationships, challenging the linear assumptions prevalent in prior research. This is consistent with the negative correlation between family size and higher PA participation reported by Jurj et al. [37]. These also align with social capital theory [47], where core networks (characterized by high density, strong ties, and homogeneity) foster expressive behaviors (e.g., maintaining exercise habits), while loose networks support instrumental actions (e.g., resource acquisition). Qualitative insights suggest two possible explanation: strong ties enhance social support for maintaining exercise routines; larger and looser networks may demand greater social engagement, reducing time available for leisure activities.

However, inconsistent conclusions exist. Research in Latin American and U.S. populations have found positive associations between network size and healthier diabetes-related behaviors [36, 48], while a Swiss study observed no link between network size and weekly exercise frequency [30]. These discrepancies may stem from: (1) cultural heterogeneity: variations in societal perceptions of network functions; (2) methodological limitations: the name-generator method relies on participant recall and artificial nomination caps, potentially underestimating true network size [49]; (3) analytical bias: linear modeling may obscure true non-linear relationships; (4) structure–function disconnect: network features directly tied to health behaviors (e.g., exercise-specific support density) may surpass basic structural metrics in predictive value. These inconsistencies suggest the need to integrate cross-cultural data, employ objective network measurements and non-linear models, and explore functionally oriented network indicators to deepen mechanistic understanding.

#### LTPA-related structural characteristics

Our study has explored functionally oriented network indicators to deepen mechanistic understanding. We found patients with higher MV-LTPA exhibited significant clustering of physically active members within social networks, independent of sociodemographic and physical health-related factors, which aligns with findings from Western studies [30, 50, 51]. Our qualitative findings further support explanations related to social contagion or social norm effects, indicating individuals’ behavioral choices are influenced by the actions and opinions of significant others [35, 52]. These insights inform intervention strategies, such as identifying “exercise advocates” within networks to leverage peer influence for habit formation, and establishing community-based exercise groups to foster a culture of PA [53, 54]. However, it is important to note that these patterns could also result from selection effects, reflecting a bias in relationship formation toward individuals with similar exercise preferences [55]. Future studies will require longitudinal data to disentangle these pathways.

#### Key role models in network-driven interventions

Current evidence is lacking on selecting specific network members for inclusion in social network interventions, particularly within China’s sociocultural context. This study identified that among middle-aged and older hypertensive patients, spouses demonstrated significant positive effects on patients’ MV-LTPA levels. These findings aligned with Maia et al. [31] on familial clustering of PA and are further supported by cross-national validation of spousal influence [56, 57], providing an evidence-based rationale for culturally adapted social network interventions. We recommended prioritizing spouses as "exercise role models" in such interventions, particularly in older populations where kinship networks predominate.

### Distinct correlates and social mechanisms of L-LTPA

Our findings extend beyond MV-LTPA to elucidate the unique socio-ecological landscape of L-LTPA, revealing behavioral profiles that are distinct in their determinants and social drivers. Unlike MV-LTPA adherence, which was associated with multiple sociodemographic factors, engagement in L-LTPA was independently predicted only by occupation and self-rated health. The higher odds of L-LTPA among farmers are consistent with the lower MV-LTPA previously reported in this population [15, 42], suggesting that high occupational exertion crowds out vigorous leisure activities while promoting lighter ones, as corroborated by our qualitative data. Conversely, the significantly lower odds among those with very poor self-rated health, which aligns with evidence linking LPA group to poorer general health [58]. It suggest that even light activities may be challenging for the most vulnerable patients, highlighting this subgroup as a critical target for supportive, low-intensity interventions.

The social network mechanisms underlying L-LTPA further demonstrate its uniqueness. L-LTPA benefited linearly from higher network density. This stable association suggests that the low-pressure environment of close-knit networks is uniquely suited to sustaining light activities, which demand less mutual reinforcement than MV-LTPA. The triphasic dynamic of LTPA aggregation—where the benefit peaked at a moderate level (approximately one-third) of active members and then declined to a steady level—is a landmark finding. This suggests that a critical mass of active peers provides optimal modeling for light activities without creating normative pressure. However, once active members exceed a majority, individuals may be propelled toward more vigorous exercise. The decline in L-LTPA likely signifies a shift to MV-LTPA, a conclusion supported by the linear increase in MV-LTPA with LTPA aggregation.

At the relationship level, the influence of network members on L-LTPA revealed nuanced patterns, even if some associations did not retain statistical significance after strict correction. The positive associations with spouses and children align with China's kinship-centric social fabric and are theoretically suggestive. The role of spouses, consistent across activity intensities, underscores their central position in chronic disease management within households. Conversely, the observed negative association with neighbors' LTPA warrants further investigation. While non-significant post-correction, it may point to unmeasured confounding factors or reveal a complex social comparison dynamic. Unlike the supportive role of family, neighbors' activity might be perceived as a source of upward social comparison or competition [59, 60], thereby failing to provide the same low-pressure support for light activities and potentially even discouraging engagement.

### Implications for practice and policy

This study identifies actionable strategies for improving LTPA among hypertensive patients: (1) Targeted interventions: Prioritize males, rural residents, farmers, and employed subgroups through community-based programs. (2) Network-driven approaches: Integrate social network mechanisms into national fitness initiatives, and leverage exercise activists within patients’ networks (e.g., spouses) as role models. (3) Policy integration: Adopt the "Exercise is Medicine" framework in primary care, empowering general practitioners to prescribe exercise plans in collaboration with sports agencies.

### Strengths and limitations

The study’s strengths include: (1) a representative baseline sample from a nationally managed hypertension cohort would facilitate future longitudinal studies and intervention trials, and (2) a mixed-methods design captured both structural and functional network dynamics. However, limitations exist: (1) Findings reflect community-based hypertensive populations in central China; patterns may differ among hospital-based populations or those residing in social contexts dominated by weak ties. (2) Cross-sectional survey precludes causal inferences; longitudinal or interventional studies are needed. (3) Self-reported LTPA may introduce recall bias and overestimation, as evidenced by prior research comparing subjective and objective measures [61]. Additionally, although individuals' behaviors are more strongly influenced by their perceptions of others' actions than by actual behaviors [62], such perceptions may not fully align with objective reality. Future studies should incorporate objective measures (e.g., accelerometer) to bridge gaps among perceived, self-reported, and actual activity data.

## Conclusions

This study investigated LTPA levels among Chinese hypertensive patients and explored the influence of social networks as well as their potential for intervention. Although LTPA levels exceed those of the general population, significant gaps persist, particularly among marginalized subgroups. Social networks represent modifiable factors that influence LTPA behaviors. By shaping network structures, fostering positive norms within networks, and strategically engaging key members (e.g., spouses), policymakers and clinicians can design culturally resonant interventions to bridge the LTPA gaps. These insights not only advance behavioral theory but also offer pragmatic solutions for global hypertension management in aging populations.

## Data Availability

The datasets used/analyzed during the current study are available from the corresponding author on reasonable request.

## Abbreviations

95% CI: 95% Confidence Interval
AIC: Akaike Information Criterion
BHPS: Basic Public Health Service
BMI: Body mass index
CDC: Center for Disease Control
edf: Effective degrees of freedom
fREML: Fast restricted maximum likelihood
GAMMs: Generalized additive mixed models
GDP: Gross Domestic Product
ICC: Intraclass correlation coefficient
IPAQ-LF: International Physical Activity Questionnaire Long Form
IQR: Interquartile range
IQV: Index of Qualitative Variation
L-LTPA: Light leisure-time physical activity
LPA: Light intensity physical activity
LTPA: Leisure-time physical activity
M-LTPA: Moderate intensity leisure-time physical activity
MV-LTPA: Moderate-to-vigorous intensity leisure-time physical activity
MVPA: Moderate-to-vigorous intensity physical activity
OR: Odds Ratio
PA: Physical activity
PHC: Primary healthcare
PPS: Probability proportional to size
V-LTPA: Vigorous intensity leisure-time physical activity
WHO: World Health Organization
